# ASSOCIATIONS BETWEEN CONTROL AND RESOLUTION OF DAILY STRESS: RESULTS FROM THE NATIONAL STUDY OF DAILY EXPERIENCES

**DOI:** 10.1093/geroni/igad104.1957

**Published:** 2023-12-21

**Authors:** Eric Cerino, Dakota Witzel, Chloe Horowitz, David Almeida

**Affiliations:** Northern Arizona University, Flagstaff, Arizona, United States; Penn State University, State College, Pennsylvania, United States; Northern Arizona University, Flagstaff, Arizona, United States; The Pennsylvania State University, State College, Pennsylvania, United States

## Abstract

Literature identifies perceived control as an important psychosocial resource for healthy aging. How perceived control over aspects of daily life influence the daily stress process, however, remains comparatively less clear. Resolution of daily stressors (e.g., an argument resolved, a leaky pipe fixed) is emerging as a characteristic of the daily stress process crucial for emotional downregulation. Using data from the National Study of Daily Experiences (N=1,766, Mage=56.25, SD=12.20, 57% Female), we examined associations between perceived control over daily stress and the likelihood of stressor resolution. In waves conducted in ~2008 and ~2017, participants reported perceived control over stressors they experience across eight consecutive days (arguments, avoided arguments, work overloads, home overloads, network stressors), in addition to whether the stressful experience has been resolved. Generalized multilevel models adjusted for linear trends across days and waves, as well as number of stressors, sex, education, race, and baseline age. People experiencing greater stressor control were more likely to resolve their daily stressors (OR=1.92, 95% CI: 1.74–2.13, p<.001). Further, individuals were more likely to resolve their stressors on days when they perceived more control over their stressors than usual (OR=1.66, 95% CI: 1.57–1.77, p<.001). This within-person association increased in magnitude across assessment waves (OR=1.21, 95% CI: 1.06–1.39, p<.01), resulting in a stronger association between stressor control and resolution 10 years later (OR=1.89, 95% CI: 1.69–2.12, p<.001). Associations did not depend on baseline age (ps>.05). Results indicate perceived control may serve as a psychosocial resource for promoting resolution of daily stressors as people grow older.

